# A Cure for Sick-Headache

**Published:** 1881-03

**Authors:** 


### A CURE FOR SICK HEADACHE.

This complaint is the result of eating too much and exercising too little. Often the cause is that the stomach is not able to digest the food last introduced into it, either from its having been unsuitable or excessive in quantity. It is said a diet of bread and butter, with ripe fruits or berries, with moderate, continuous exercise in the open air sufficient to keep up a gentle perspiration, would cure almost every case in a short time. To drink two teaspoonfuls of powdered charcoal in half a glass of water generally gives instant relief. The above sovereign remedies may do in some, but not in all cases. A sovereign remedy for this ailment is not easily found. Sick headache is periodical, and is the signal of distress which the stomach raises to inform us that there is an over-alkaline condition of its fluids; that it needs a natural acid to restore the battery to its normal working condition. When the first symptoms of a headache appear, take a teaspoonful of clear lemon juice fifteen minutes before each meal and the same dose at bedtime; follow this up until all symptoms are passed, taking no other remedies, and you will soon be able to go free from your unwelcome nuisance. Many will object to this because the remedy is too simple, but many cures have been effected in this way. *Boston Transcript.*

### HAPPINESS.

Man, says Dumas, is always seeking after happiness; but happiness is relative and depends upon temperament. character and circumstances. There are for man only two involuntary misfortunes, misery and malady. Outside of these congential fatalities, what he calls his misfortune is always his own work. Life does not realize all his hopes, and then he declares himself to be unfortunate. "In reality, man's unhappiness reduces itself to this: that he has not been as happy as he expected to be, as he thought that he had a right to be * * *. That which makes the unhappiness of the human being, always excepting native misery and malady, is that he places his happiness in perishable things which, while becoming disaggregated by the law of exhaustion and metamorphosis, leave in void stupor and despair those who have put their trust in them. Every human being who shall attach himself only to eternal things will not know that unhappiness. Hence comes that serenity of great religious men and of great philosophers; hence their benevolent, charitable and gentle contempt for human misfortunes, the cause of which they have found in the errors and weaknesses of the little human desire. There are no deceptions, no fatalities, no recriminations for those who devote themselves to the exclusive love, without calculation and terrestrial ambitions, of nature, God, art, science, humanity."—*Home Journal.*

---

### SUNBEAMS.

Time is full of new wrinkles.

The divorce lawyer's favorite fruit—a tart pair.

As the pen is bent, the paper is ink lined.—*Salem Sunbeam.*

"Be composed," as the type-sticker said to the copy.—*Cin. Sat. Night.*

A glimpse of the future—Looking down the barrel of a shot-gun with a crazy man at the other end.—*Keokuk Gate City.*

If you've made a resolution not to smoke, don't break it. But if you are offered a cigar, why, take it, and pass it around this way.—*Fulton Times.*

One of the most exasperatingly humiliating moments of a man's life comes at about 2 A. M., when he gets on his front door stoop and finds that his night key is in his other vest.—*Newark Sunday Call.*

A Galveston colored servant, on her way home with a basket of pilfered groceries, meets a friend. "How is you comin' on wid dem white folks?" "I'se gwine to leab 'em. Dey hasn't paid dar groceryman in sich a long time I'se ashamed to meet him on de street."

Carving isn't fun. A young man was invited to carve a turkey at dinner, recently, and before the knife was finally taken from him he had upset a glass of water, wrenched his shoulder, shot the bird across the table into a lady's lap, and nearly jabbed a man's eye out, and it wasn't a tough bird, either.

"Did you slip?" they asked the old gentleman, as they picked him up. "Oh, no," he growled, "of course not. I was trying to see if I could sit down on that coal-hole top hard enough to break it. Did it just for the fun of the thing." And he glared at them savagely, and they somehow felt mighty foolish.—*Boston Post.*

---

One day, during an eclipse of the sun, a boy sold smoked glasses at six cents apiece. "You ought to make money," said a purchaser. "Yes," said the young merchant, "ours would be a good business if the dull season were not so long."

### AN AUTOCRAT'S HUMOR.

The late Emperor Nicholas happened one day to be engaged in inspecting a State penitentiary in one of the provincial seats of government, and took it into his head to question some of the convicts respecting the nature of the offences for which they were suffering punishment. "What are you here for?" he asked of one.

"I am innocent, Imperial Majesty," replied the prisoner, falling on his knees; "a victim of false witness! A church was robbed—a beadle knocked on the head—the peasants caught hold of me, and I knew nothing about it!"

Similar replies were given by other prisoners. The Emperor, obviously bored by these successive protestations of guiltlessness, cast a glance along the line of prisoners until his eye fell upon a ragged, wretched-looking gypsy, whom he beckoned forward with the words:

"Of course you, too, are here on a false charge?"

"Not a bit of it, your Majesty!" replied the Tsigan; "It is all fair and square as far as I am concerned. I stole a pony from a tradesman."

"Stole a pony, did you?" said the Czar, with a laugh; and then, addressing the governor of the prison with well-assumed sternness: "Turn that good-for-nothing rascal instantly out of doors. I cannot allow him to remain a minute longer in such honorable and virtuous company, lest he pervert all these good, innocent people."

---

A SHREWD little fellow lived with an uncle who barely afforded him the necessaries of life. One day the two were out walking together, and saw a very thin greyhound, and the man asked his nephew what made the dog so poor. "I expect he lives with his uncle," said the boy.

A TEXAS editor states that a negro in Houston County, that State, made $30 last fall in picking cotton after gathering his own crop. He and his wife had previously made ten bales clear of all expenses. He owes nothing and had $500 in the bank. He now intends to purchase a place for $1,200, paying his $500 down, and expects to make enough next year to pay the remainder. The editor who describes this says that about the time that the negro was negotiating for the property, four or five hearty, strong, young white men came to the writer's house begging for food.

### LIFE INSURANCE WITHIN THE REACH OF ALL.

INSURING one's life, which most people deem a duty, has hitherto been a luxury too costly for people of moderate means. Life insurance companies, in order to accumulate their enormous surplus funds, in many cases amounting to millions of dollars, have fixed their rates of premiums at figures which, we repeat, are prohibitory.

The extravagance of what we may properly call this old system of insurance has driven great numbers of persons to the adoption of a newer and better system of life insurance, within the reach of those to whom it is of the greatest use, to wit, persons of moderate means. This system is strictly mutual, and therefore the safest of any ; since, after providing for the ordinary expenses of economical management, members are only taxed the small sums required from time to time to make up the losses by death ; thus members are not taxed three or four times as much as is necessary in order to establish an enormous surplus fund in which they have no interest beyond the amount of their insurance. We believe in the new plan of life insurance, and have shown our faith by taking policies of insurance in two associations. We will forward all necessary printed information to any reader of the JOURNAL who will take the trouble to write to us on the subject.

**KNIGHT BROTHERS,**

SOLICITORS OF

UNITED STATES AND FOREIGN

**PATENTS,**

**Counsellors in Patent Causes**

AND

**MECHANICAL EXPERTS,**

AGENCIES AT PRINCIPAL FOREIGN CAPITALS.

**No. 633 F Street, N. W., Washington, D. C.;**
**75 West Fourth Street, Cincinnati, Ohio;**
**317 Olive Street, St. Louis, Mo.**

**SEND FOR BOOK OF INSTRUCTIONS.**

### DR. HALL'S PORTABLE SPIROMETER.

NO PHYSICIAN questions the value of the spirometer for exercising and expanding the lungs, and for measuring their capacity. The great difficulty has been to find an instrument that can be compressed into a small compass when not in use, so that the physician or patient may conveniently carry it from place to place as circumstances require. Another obstacle to the more general use of this truly valuable instrument has been its price. The unwieldy spirometer of a few years ago cost from $10 to $20. Dr. Hall's Portable Spirometer sells for $7.50, and will be sent free to any address on receipt of the price by the editor of this journal.

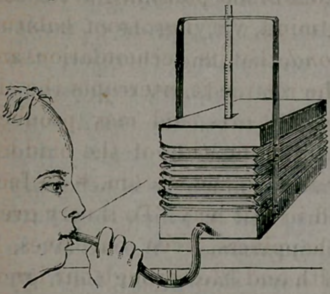

It is 6 by 12 inches, and when closed it stands only 3 inches high, and weighs less than 3 pounds.

This cut represents Dr. Hall's Portable Spirometer, partly inflated, in order to show the manner of using it. When not in use it can be closed together so as to occupy the small space above stated. The scale and guide are hinged, so that they close down on the top of the spirometer, and occupy but little space. The top is of polished black walnut. The brass-work is plated with silver or nickel.

HOW TO USE THE SPIROMETER.

The instrument should be used only in a perfectly ventilated room, which is entirely free from dust, or else in the open air, away from the dust and smoke and bad odors of the streets of a city. When it is remembered that the capacity of the lungs of a man of medium size is 150 to 250 cubic inches, and that in the act of breathing naturally, or rather as some have acquired the habit of breathing—which is far from natural—the lungs take in only from 20 to 40 cubic inches of air at each inspiration, leaving from 130 to 230 cubic inches of air cells almost always in a dormant condition, the importance of regular and systematic exercise for the lungs will be appreciated. But, like all good things, this sort of lung exercise must be indulged in moderation. Draw a full breath and then blow through the rubber tube into the spirometer; at the same time watch the figures on the scale as it rises. The figures seen on the scale as it passes through the top of the guide indicate the number of cubic inches of air blown into the spirometer.

It is now very generally conceded that mankind start quite evenly on the journey of life, in spite of supposed hereditary tendencies to pulmonary consumption, and that with proper care the average term of life is not shortened by these hereditary tendencies. This being the case, it is far better to enjoy health and long life through means which the naturally robust are not compelled to resort to, than to believe in or lament over the inheritance of ills that do not exist. We believe for all persons who are confined much of the time in doors, particularly in cases where there is a tendency to weak lungs, the regular use of the spirometer in the open air is highly beneficial. Its use just before bedtime is said to induce sleep, by drawing blood from the brain to the lungs.

### CONSTIPATION.

CONSTIPATION becomes alarmingly frequent as people acquire the means of living luxuriously without regular manual labor. It is still more frequent among persons who become so through ignorance of the penalties that follow a want of attention to the regular demands of nature.

When fecal matter is retained in the rectum more than twenty-four hours, it commences its destructive work, notably in two ways. *First*, It is absorbed into the system, and acts as a blood poison. One of the worst results from blood poisoning from constipation is to cause disease of the mind, varying from habitual moroseness to actual insanity. *Second*, By its accumulation and impacting in the lower portion of the rectum it interrupts the circulation of the blood in the adjoining parts, and may produce, from this cause alone, piles, fistula, inflammation of the bladder, disease of the prostate gland, or cancer of the rectum. In fact, most of these diseases are developed in this way. To a very great extent we hold our health and happiness, even our lives, in our own hands. If we sacrifice health and life through our ignorance, or indifference, we pay the penalty through suffering, more severe because more prolonged than instant destruction. From whatever cause, there is, unfortunately, great ignorance in regard to the laws of health among a class of people who are otherwise intelligent. How many parents are there who look carefully to the habits of their children, upon which all that will make life desirable for them so much depends? Cathartics and clysters should not be used in constipation. They increase the trouble.

That which has, perhaps, met with most general favor relates to the diet. Certain foods have been regarded as specifics for this complaint, and particularly "Graham bread." This often induces activity of the bowels, but it ruins digestion like other cathartics, in a manner which we have heretofore fully explained in the pages of this journal. Kneading the bowels has sometimes afforded relief, but it affords no permanent benefit, as a rule. We have, however, found a remedy for constipation and for piles which is always safe, and is attended with no inconvenience or discomfort. We have prescribed it in hundreds of cases. It has usually afforded prompt relief, and many patients who have suffered for years believe that they have been permanently cured of constipation by its use. This remedy is "Nelaton's Suppository." It is prepared and sold by Hall & Ruckel, wholesale druggists, 218 Greenwich street, New York, at 50 cents and $1.00 a box, and is sent free by mail on receipt of price.

